# Supplementary material for: Adequacy of the WHOQoL-BREF to Assess the Quality of Life of Victims of Armed Conflicts
Source: Assessment. 2023 Oct 30;31(5):1135–47. doi: 10.1177/10731911231201145 (PMC11134973; doi:10.1177/10731911231201145)
Supplement: sj-docx-1-asm-10.1177_10731911231201145 – Supplemental material for Adequacy of the WHOQoL-BREF to Assess the Quality of Life of Victims of Armed Conflicts [file sj-docx-1-asm-10.1177_10731911231201145.docx]

**Abstract**

There is a deterioration in the quality of life (QoL) of survivor victims of warlike conflicts. Because there is a need to guarantee the effectiveness of assessment tools for these populations, we studied the adequacy of the World Health Organization Quality of Life Questionnaire (WHOQoL-BREF) to assess the QoL of 1136 surviving victims of the armed conflict in Colombia. Although this questionnaire has yielded promising results, questions remain about its psychometric suitability for specific populations. We used model modification at the item level, comparisons of models with different factor structures, and dimensionality analysis to address the psychometric problems encountered. Dimensionality analysis using a bifactor model suggests that WHOQoL-BREF total scores might be a more appropriate way of reporting results when model' fit adequacy is not reached. Conclusions are offered on the psychometric properties of the WHOQoL-BREF, the evaluation of special populations, and possible strategies to address future questionnaire modifications.

**Keywords**: quality of life; WHOQoL-BREF; victims; factorial analysis; bifactor model.

**Introduction**

Quality of life (QoL) is the "individuals' perceptions of their position in life in the context of the culture and value systems in which they live and concerning their goals, expectations, standards and concerns" (WHO, 1996, p. 5). The study of QoL has gained more relevance in recent years due to a broadening in focus, involving the measurement of health beyond traditional health indicators such as mortality and morbidity (WHO, 1996). However, QoL could have different meanings according to the area of application and this is reflected in the evaluation instruments. Some QoL measurements focus on a single concept, for example, emotional functioning; while others assess individual concepts, such as QoL dimensions (Fayers & Machin, 2001). The latter understand QoL as a multidimensional construct.

One of the most widespread questionnaires on QoL is the WHOQoL-BREF (WHO, 1996). It is the abbreviate 26-item version of the WHOQoL-100 (The Whoqol Group, 1998; WHO, 1996). Although it was initially designed and used for healthy populations (Hargreaves et al., 2021; Masaeli & Billieux, 2022), it has also been used to evaluate special (Gagliardi et al., 2021; Singh et al., 2022), and clinical populations (Guaracha-Basáñez et al., 2022; Martini et al., 2022). By special population we refer to populations not classified as clinical but who have experienced significant contextual situations that drastically affect their QoL, for example, long-term caregivers, refugees, war survivors, and more.

Contradictory results have been reported on whether WHOQoL-BREF is a psychometrically suitable measure for all populations. Lucas-Carrasco (2012) reported successful use of the WHOQoL-BREF to assess psychiatrically and physically affected populations, as well as healthy populations. On the contrary, other authors (Benitez-Borrego et al., 2014; López Huerta et al., 2017; Ohaeri et al., 2007; Oliveira et al., 2016) found psychometric issues with the WHOQoL-BREF when evaluating similar populations.

Given the current world scenario where multiple armed conflicts affect people's lives, there is a need to ensure tool effectiveness when assessing these populations. More effective studies on the psychological impact of war will support the development of better strategies to improve the victims' QoL. This is particularly relevant in victims of the armed conflict in Colombia (Hewitt-Ramírez et al., 2020) where low to medium QoL values have been reported (Simancas-Fernández et al., 2021).

To our knowledge, the WHOQoL-BREF has not been fully assessed in terms of psychometrics for the specific situation of victims of armed conflicts. Thus, the present study’s aim was to evaluate the use the WHOQoL-BREF to report QoL in the victims of armed conflict in Colombia.

**Psychometric Issues of the WHOQoL-BREF**

QoL, evaluated by the WHOQoL-BREF's manual, comprises four domains: physical, psychological, social relations and environment. The extended 100-item version of the questionnaire (WHOQoL-100) includes two more domains: Level of independence and Spirituality. The factorial structure of the WHOQoL-BREF has been explored because the results persistently suggest a poor model fit. For example, Yao et al. (2002) and Vinaccia et al.(2006) found factorial discrepancies with the 4-factor structure when studying Taiwanese and Colombian samples, respectively.

Oliveira et al. (2016) found a 5-factor model more suitable for a psychiatric sample as the original model raised concerns at the factor and item levels. Additionally, these authors excluded items 12-14 and 24-26 from the WHOQoL-BREF due to low loadings.

Ohaeri et al., (2007) found evidence of a 5-factor structure, but in this case, without excluding any items. However, after model comparison, the authors concluded that the original 4-factor was a better fit. Both Oliveira et al. (2016) and Ohaeri et al., (2007) found clues of a fifth factor analogous to the Level of Independence factor in the WHOQoL-100.

Benitez-Borrego et al. (2014) explored the structure of the WHOQoL-BREF in a sample of Spanish-speakers, including analyzes of cross-country effects. They found the 4-factor structure to be the most appropriate, although the composition, that is, the items within each factor, differed from the original configuration.

QoL is usually described as a hierarchical multidimensional construct composed of four dimensions and a general factor (Lee et al., 2005) (or six dimensions, if the questionnaire extended version is used), but fit problems of the WHOQoL-BREF could be due to the assumption of dimensionality. It has been described that a bifactorial structure might be suitable in response to WHOQoL-BREF fit issues. Perera et al. (2018) found that a bifactor structure of QoL provided an excellent fit to the data when comparing to a correlated four-factor model and a high-order model.

One of the key uses of bifactor models is to determine the adequacy of a total score and what can be gained by scoring the domains; for instance whether the items are measuring the specific dimensions they are supposed to measure or whether they are just re-measuring the construct (Rodriguez et al., 2016). If adjustment problems persist, the use of total scores could be an alternative to continue using the WHOQoL-BREF even when the factor distribution does not meet psychometric standards.

All this together suggests that a deeper analysis of the properties of the model could benefit the future use of WHOQoL-BREF with special populations.

**Model Modifications**

Poor model fit and low explanatory power may indicate that the instrument, in this case, the WHOQoL-BREF, is not effectively capturing the goal construct (Weston & Gore, 2006). Model modifications are an alternative that allows researchers to use instruments despite model fit problems. However, model modifications should be made with caution and only when the modifications are theoretically and practically plausible (MacCallum, 1995). We must consider that the WHOQoL-BREF is a well-known and widely used instrument. Post hoc modifications often result in the estimation of data-based models that affect replicability (Weston & Gore, 2006). To avoid this, modifications should go from the least intrusive modification at the item level, to the factorial level, and finally to the dimensional level.

The Modification index (MI) or Lagrange multiplier is often used for model modification at the item level. MI provides an estimated value at which the model's chi-square test statistic (*χ* ^2^) decreases if a fixed parameter is added to the model and it is freely estimated (Whittaker, 2012). The process is repeated until the addition of any fixed parameter does not significantly reduce the model’s *χ* ^2^ or until none of the statistically significant potential respecifications are theoretically plausible to include in the model (Bollen, 1989).

To address model modifications at the factorial level, multiple models from the literature and/or exploratory analyzes can be set up to compete considering their ancillary fit indices (Jackson et al., 2009). For example, Benitez-Borrego et al., (2014), Ohaeri et al., (2007) and Oliveira et al., (2016) used a combination of EFA and CFA to explore new factorial structures of the WHOQoL and decide whether a different model could be more suitable. The *χ*^2^ assesses the fit between the hypothesized model and data from a set of measurement items (the observed variables) (Alavi et al., 2020). The Tucker-Lewis Index (TLI) is the proportion of total information explained by a model (Schermelleh-Engel et al., 2003). The Comparative fit index (CFI) corrects a previous basic-flow index (Bentler, 1992). The Standardized Root Mean Square Residual (SRMR) is the square root of the difference between the residuals of the sample’s covariance matrix and the hypothesized model (Hu & Bentler, 1999). Finally, the root mean square error of approximation (RMSEA) is the amount of variance not explained by the model-given degrees of freedom (Steiger, 2007).

At the dimensionality level, bifactor models provide an alternative to hierarchical QoL models (Chen et al., 2006), and they are increasingly being used in the psychological field because they provide researchers with a functional and structural representation, and are a valuable psychometric tool (Hammer et al., 2018; Rodriguez et al., 2016). Bifactor models can be used to determine the relevance of including dimension scores in the analysis or just the total score.

The feasibility of using only a total score or including dimension scores when interpreting the measure is assessed using auxiliary indices. Explained common variance (ECV) is the proportion of all common variance explained by the general factor (QoL in the present study), while for specific factors it is ECV_S and computes the strength of the factor relative to all explained variance only of the items loading in that specific factor (Stucky & Edelen, 2014). Item explained common variance (IECV) asses unidimensionality at the item level, as it measures the extent to which an item’s response accounts for the variation on the latent general dimension alone (Stucky et al., 2013). Overall reliability is informed by omega (ω) and for internal reliability omega hierarchical (OmegaH), and relative omega. OmegaH reflects the percentage of systematic variance in raw scores that can be attributed to individual differences in the general factor. Relative omega is OmegaH divided by Omega. Relative omega for the general factor is the percentage of reliable variance in the model due to the general factor. For specific factors, it represents the proportion of reliable variance in the factor that is independent of the general factor (Dueber, 2017). Replicability (H) is a measure that represents the correlation between a factor and an optimally weighted item composite. The percent of uncontaminated correlations (PUC) is the percentage of the covariance. Together with ECV, PUC influences the parameter bias of the unidimensional solution.

**Present Study**

We assessed whether the WHOQoL-BREF is psychometrically suitable to evaluate the QoL in victims of armed conflict in Colombia. We planed the modifications following a “bottom-up” analysis, aiming to preserve the original model configuration as much as possible.

We started modifications at the item level, up to the factorial structure, and ending in the dimensionality analysis. To avoid data-driven results, we restrained ourselves from omitting items; and only contemplated the alternative model if it showed meaningful improvement when compared with the original one.

We hypothesize that we will find the same WHOQoL-BREF psychometric problems previously reported in the literature. In addition, alternative models will improve the issues at the item and factor level. Finally, we prevent that the dimensionality analysis will lean towards the total score of the WHOQoL-BREFF as a more appropriate way of reporting the QoL of the population studied.

**Method**

**Participants**

The WHOQoL-BREF was administered to a sample of 1139 survivor victims of the Colombian armed conflict. The database was handled using listwise deletion, so we only performed the analysis on participants with the complete observations. The total of valid cases was 1136; 805 (70.86%) females and 331 males. At the time of the study, participants' age and education level were as follows: *M*_age_ = 42.51, *SD* = 15.39, *Range* 16 *–* 85; 362 (31.90%) had non-formal education, 378 (33.30%) had primary education, 302 (26.60%) had secondary education, and 94 (8.30%) had higher and technological education.

**Ethics**

This work is part of a research project on psychological wellbeing, QoL and social support in the victims of the armed conflict in Colombia. This project was developed by the Neurocognition Research Group (ref. COL0052341), in the framework of the project financed by CONADI- Universidad Cooperativa de Colombia (Cod.1800). It was also part of a research project on assessment of memories and psychological disorders associated to trauma in refugees and victims of war, developed by the UCM Research Group on Eyewitness Testimony (ref. 971672), in the framework of the project financed by Santander-Universidad Complutense de Madrid (PR87/19-22576).

**Materials**

Participants were assessed with the abbreviated Spanish version of the WHOQoL developed by WHO (WHOQoL-BREF, 1996). This Likert-type cross-cultural questionnaire asks for satisfaction on 26 items. The first two items (Q1 and Q2) refer to general health and general QoL and they should be analyzed independently according to the WHOQoL-BREF’s manual. Participants should answer the other 24 items (from Q3 to Q26) concerning the last two weeks of their life. QoL scores are distributed among four domains for interpretation purposes: physical health (pain and discomfort, dependence on medication or treatment, energy and fatigue, daily activity, mobility, sleep and rest, and ability to work); psychological health (positive feelings, spirituality, religion and personal beliefs, thinking, learning, memory and concentration, personal image and appearance, self-esteem and negative feelings); social relationships (personal relationships, sexual activity, and social support); and environment (physical security and protection, physical environment, economic resources, opportunities for information/skills acquisition, home, health and social care, and transportation). The results are reported as total scores and scores by domains.

**Procedure**

We ran a CFA following the structure suggested by the WHOQoL-BREF’s instructions to evaluate the adjustment of the original model structure to the current population. Ancillary fit indices indicated whether we had to proceed with model modifications.

We proceed to cross load items based on modification index score values (MI). Modifications were done sequential and incrementally starting with the highest MI; we kept it if the model fit improved, and check for new modifications.

We compared the modified 4-factor WHO’s structure with the 5-factor structure proposed by Oliveira et al. (2016) and the 5-factor structure proposed by Ohaeri et al. (2007). The 6-factor structure proposed by Benítez-Borrego et al. (2014) was originally included but resulted in an unidentified model.

Finally, we compared a hierarchical model of QoL with alternative unidimensional and bifactor models. These analyses will determine whether a total score of the WHOQoL-BREF is more suitable than using the factors as subscales.

**Data analysis**

We performed the analyses using RStudio (version 4.0.2.) and we use the Dueber’s calculator tool (Dueber, 2017) to assess ancillary indices of the bifactor model.

For cross loading the items, we sought fixed parameters associated with a large MI value (i.e., larger than a *χ* ^2^ critical value of 3.84, which corresponds with 1 degree of freedom at an alpha level of .05) to decide whether they would be theoretically plausible to include in the model and be freely estimated (Whittaker, 2012). We ended up with a modified 4-factor WHO’s structure.

The decision on what model should be conserved was taken according to fit indices and other statistics, conservation of the latent variables, and the avoidance of data-specific results during model comparisons. We followed the recommendation of using statistical indices that reflect different properties of the structures as a threshold for model fitting (Kline, 2005). The optimal values of model ancillary indices were considered as comparative parameters to determine which of the competing models is the best option (Bentler, 1990; Bollen & Bauldry, 2011; Hu & Bentler, 1998, 1999; Widaman & Thompson, 2003; Yuan, 2005). Indices cutouts indicate a good fit if the TLI value is 0.97 or superior (Schermelleh-Engel et al., 2003); the CFI value is greater than 0.90 (Bentler, 1992), the SRMR smaller or close to 0.08 (Hu & Bentler, 1999); and the RMSEA value is less than 0.07 (Steiger, 2007) or 0.06 (Hu & Bentler, 1999).

We assessed dimensionality through ancillary indices as suggested by Dueber (2017). Many IECV with values greater than 0.80 or 0.85 typically indicate unidimensionality (Stuckey & Edelen, 2015). If OmegaH is over 0.80, total scores can be considered unidimensional (Reise et al., 2013). Replicability values over 0.80 suggest a well-defined latent variable (Hancock & Mueller, 2001). When ECV and PUC are over 0.70, relative bias will be slight and the common variance can be regarded as essentially unidimensional (Rodriguez et al., 2016). If PUC values are lower than 0.80, general ECV values are greater than 0.60, and OmegaH of the general factor is above 0.70, the presence of multidimensionality is not severe enough to disqualify the interpretation of the instrument as unidimensional (Reise et al., 2013).

**Results**

**WHOQoL-BREF adjustment**

Descriptive information of the total sample appears in Table 1. Distribution analyses indicated data close to normal (skewness [-0.23 – 0.65], kurtosis [-1.00 – 0.63]) for the overall sample. Correlations among items were below 0.51, so they did not raise concerns about overlapping items.

Table 1 here

The CFA model that follows the structure suggested by the WHOQoL-BREF’s instructions showed a significant chi-square goodness of fit test *χ* ^2^ (246, *N* = 1136) = 829.638, *p* < 0.001, suggesting poor model fit. Fit indices showed a relatively small proportion of total information explained by a model (TLI = 0.948), a relatively high parsimony corrected index (RMSEA = 0.046; 90% CI: [0.42-0.49]), a moderate mean absolute residual correlation SRMR (0.055); and a relatively low improvement of the model over another that assumes uncorrelated indicators value (CFI = 0.954). Factors showed low reliability values: physical (α = 0.672, ω = 0.659), psychological (α = 0.704, ω = 0.713), social relations (α = 0.630, ω = 0.634), and environment (α = 0.709, ω = 0.691).

Items Q3 (0.24), Q4 (0.24), Q12 (0.40), Q14 (0.40) and Q26 (0.37) showed low loadings into their respective dimensions. Factors psychological and social relations (r =.80), psychological and physical (r = .88), social relations and physical (r = .72) were highly correlated (see Supplementary material 1). All these suggest some modifications could significantly improve the model.

**Modification indices adjustments**

We cross loaded the item Q8 *How safe do you feel in your daily life?* (MI = 168.09) from the environment domain on the psychological domain; and Q9 *How healthy is your physical environment?* (MI = 55.84) from the environment domain on the psychological domain. Because of theoretical and practical reasons, we decided to stop cross load modifications after Q9. We did not include the next highest MI score that suggested that Q22 *How satisfied are you with the support you get from your friends?* (MI = 30.18) from the social relations domain should be cross loaded on the environment domain (see Supplementary material 2). The fit indices improved minimally and adding more modifications would continue to decrease the degrees of freedom of the model. As can be observed in Table 2, the model fit indices slightly improved after item modifications.

Table 2 here

The correlation between psychological and social relations (r = .79) psychological and physical (r = .86) reduced slightly. The correlation between physical and social relations dimensions remained as it was (r = .72).

Factor physical reliability remained as it was in the original model (α = 0.672, ω = 0.659). There were small reliability improvements in the Psychological (α = 0.746, ω = 0.744), and social relations (α = 0.630, ω = 0.635) factors. Reliability of the environment factor slightly decreased (α = 0.709, ω = 0.678).

MI values also suggested a relevant error covariance between Q3 and Q4 (MI = 91.05), between Q24 and Q25 (MI = 44.43), between Q23 and Q24 (MI = 26.21.80), and between Q26 and Q12 (19.08). No modifications derived from these, but the error covariances will be addressed later in the discussion.

**Model comparisons**

Table 3 summarizes the values of the fit indices of the three models to be compared: A 5-factor model (5F) (Ohaeri et al. 2007), a reduced 5-factor model (5F_R_) (Oliveira et al. 2016), and the 4-factor model (4F) derived from the previous section’s analysis.

Table 3 here

As observed in Table 3, 5F_R_ showed some improvement in all the indices compared to 5F and 4F. However, we must consider that this model has fewer items, and despite its indices and degrees of freedom improved due to fewer items, Chi-square was statistically significant. Additionally, we did not find strong enough evidence to exclude Q24, Q25, Q12, Q13, Q14, Q26 from the model. We decided to dismiss the 5F_R_ model as we aimed to preserve the original model as much as possible and the improvement did not justify leaving that many items out of the model. The 5F showed no improvement over the 4F.

**Dimensionality analysis**

QoL is described as a hierarchical multidimensional construct composed of four dimensions and a general factor (Lee et al., 2005). The hierarchical, unidimensional and bifactor models fit were compared. Table 5 and figures 1, 2, and 3 show a summary of the hierarchical, unidimensional and bifactor models.

Table 5 here

Figure 1 here

Figure 2 here

Figure 3 here

As shown in Table 4, the bifactor model showed better fit than the hierarchical and the unidimensional models; suggesting that the QoL model could be improved if a different dimensionality structure is assumed.

The standardized loadings of the unidimensional model and the bifactor solutions appear in Table 5. The bifactor model fit indices showed the best adequacy of all the tested models: *χ* ^2^ (226, *N* = 1136) = 420.113, *p* < 0.001; TLI = 0.981, RMSEA = 0.028, 90% CI: [0.023-0.032]; CFI = 0.985; SRMR = 0.038 (see Supplementary material 3). When bifactor models fit, ancillary bifactor measures must be consulted.

Table 5 here

To support the use of the unidimensional model in the evaluation of QoL in the present sample, we consulted bifactor measures pertaining to model-based reliability of the total score and the subscale scores. At the item level, 76.92% of the items showed item explained common variance (IECV) above 0.50, and 50% of the items showed IECV values above 0.80 suggesting that items were largely accounted for by variation on the general dimension alone (Stucky et al., 2013). The explained of the factor common variance for specific factors (ECV_S) for the physical ECV_S = 0.12, for the psychological ECV_S = 0.07, for social relations ECV_S = 0.05, and for the environment factor ECV_S = 0.16. Finally, the general factor QoL explained common variance (ECV) was ECV = 0.61.

Reliability analysis showed that 79.30% of the variance of the total scores could be attributed to the individual differences on the general factor (OmegaH = 0.79). All the reliable variance of the total scores could be attributed to the general factor of QoL (relative omega = 0.90). Only around 10% (ω = 0.89 – OmegaH = 0.79) of the reliable variance in the total scores could be attributed to multidimensionality caused by the factors.

The construct replicability measure showed that the general factor is a well-defined latent variable, H = 0.88. The factors’ construct replicability showed less latent variable definition: physical, H = 0.87, psychological, H = 0.45, social relations, H = 0.34, and environment, H = 0.68.

The percent of uncontaminated correlations (PUC) was above 0.70 (PUC = 0.75), and ECV was close to 0.70 (ECV = 0.61). We can consider the relative bias as slight and the common variance can be considered as essentially unidimensional (Rodriguez et al., 2016). Additionally, since the PUC value was lower than 0.80, the general ECV was greater than 0.60 and the OmegaH of the general factor was higher than 0.70; it is suggested that the presence of some multidimensionality is not severe enough to disqualify the interpretation of the instrument as unidimensional (Reise et al., 2013).

**Discussion**

Our results suggest that the study of QoL in special populations can benefit from considering this construct as unidimensional. Even though the literature refers to QoL as a multidimensional construct composed by four latent variables (Lee et al., 2005); we found evidence of the unidimensionality of QoL in the present sample. We also confirmed some psychometric problems with the WHOQoL-BREF previously reported in the literature. By no means our results oppose the factorial structure reported in the WHOQoL-BREF manual. We suggest that when the factorial structure does not meet the expected fit for the sample, researchers can still benefit from using total scores. Even when the items are not successfully capturing their specific factors information, or factors are not successfully related to the general construct, the items are still effectively evaluating QoL in the form of total scores.

One thing to consider is whether special conditions affect the configuration of the construct. In other words, whether self-perceived QoL might differ depending on, for example, whether the person experiences prolonged trauma, compared to the experience of the general population. We believe this difference could be reflected in dimensionality variations of the latent variables of the construct.

This study was carried out with a population that, we suspect, might have an atypical perception of QoL. This could be why the QoL model found in the general population does not fit our data. The unidimensional behaviour of QoL in the present study raises questions about the effectiveness of the use of instruments not initially designed for special populations, especially considering that the factorial structure is part of the interpretation of the results in the form of subdomains. In general, we recommend considering whether subdomain analyzes are applicable when using the WHOQoL-BREF in cases where QoL perception alterations are suspected.

Our results may also be worthy of consideration in future revisions of the WHOQoL-BREF. As we confirmed, the reported problems persist at the item and factor level. Some of these are discussed in the next section.

**Future psychometric considerations**

A concern in the present study was obtaining sample-specific results that would affect future replicability in similar samples. Although population-tailored models are valuable for understanding variations within populations, replicability and validity remain critical. In our study, we leaned towards preserving the items and the factorial structure, mainly because our modifications did not justify major modifications of a well-established model.

However, we must highlight that the MI values consistently suggested a relevant error covariance between Q3 and Q4, indicating that there is a systematic variance shared by these items that is not explained by any factor. This matches the results found by López Huerta et al. (2017) and Theuns et al. (2010) who suggested that further analysis should consider the contribution of Q3 *To what extent do you feel that physical pain prevents you from doing what you need to do?* and Q4 *How much do you need any medical treatment to function in your daily life?* to the QoL model. From all the items originally within the physical (Q10, Q15, Q16, Q17, and Q18) domain, these two are the only ones that explicitly ask about medical treatment and pain. The rest refer to energy and capabilities that may have different origins, not only medical, and may be more associated with QoL in populations without specific health needs.

The inclusion of item Q26 *How often do you have negative feelings such as blue mood, despair, anxiety, depression?* also appears to be debatable because it showed low loadings in all models. Even though psychological disorders are relevant for QoL perception, we must consider that there are many potential QoL dimensions and it is impractical to try to assess all these concepts simultaneously in one instrument (Fayers & Machin, 2001).

Based on modification indices, we chose to load Q8 and Q9 into the psychological and environment domains. As for how this change would affect the theoretical interpretation of the domains, Q8 asks *How safe do you feel in your daily life?* And it could also be interpreted as also psychological as the "feeling of safety". Q9 asks *How healthy is your physical environment?* The item explicitly asks about the physical environment. However, a "healthy environment" could also be interpreted as psychological as in a "working environment." We cannot leave aside that some properties of the items may change due to translation, evaluation conditions, or cultural differences (Theuns et al., 2010).

Future studies may approach these item-level issues by testing models that leave them out or modify them. We decided to preserve all the items because validation was not the scope of our study and even items with low loadings might be capturing information about the construct.

**Conclusions**

The use of instruments validated in healthy populations to evaluate special populations is a common practice in Psychology. Even though instruments can benefit from the diversity resulted from this practice, special populations may be misrepresented by those measures. QoL responses refer to subjective perceptions that may be affected by life events, such as prolonged stress and trauma. In conclusion, we appeal to the careful use of measures not designed to assess significantly affected populations. This could be of particular interest to researchers who aim to study and help lessen the psychological impact of war.

An alternative to being able to use validated instruments with model fit problems is to consider whether researchers can benefit from using total scores instead of dimensions that may not reflect the behavior of the construct in special populations. We thoroughly considered models that assumed different dimensionalities and given the persistence of item and factor level issues, we explored the viability of using total scores. As we found, there is evidence that researchers can benefit from using total scores.

**References**

Alavi, M., Visentin, D. C., Thapa, D. K., Hunt, G. E., Watson, R., & Cleary, M. (2020). Chi‐square for model fit in confirmatory factor analysis. *Journal of Advanced Nursing*, *76*(9), 2209–2211. https://doi.org/10.1111/jan.14399

Benitez-Borrego, S., Guàrdia-Olmos, J., & Urzúa-Morales, A. (2014). Factorial structural analysis of the Spanish version of WHOQOL-BREF: An exploratory structural equation model study. *Quality of Life Research*, *23*(8), 2205–2212. https://doi.org/10.1007/s11136-014-0663-2

Bentler, P. M. (1990). Comparative fit indexes in structural models. *Psychological Bulletin*, *107*(2), 238–246. https://doi.org/10.1037/0033-2909.107.2.238

Bentler, P. M. (1992). On the fit of models to covariances and methodology to the Bulletin.. *Psychological Bulletin*, *112*(3), 400–404. https://doi.org/10.1037/0033-2909.112.3.400

Bollen, K. A. (1989). A New Incremental Fit Index for General Structural Equation Models. *Sociological Methods & Research*, *17*(3), 303–316. https://doi.org/10.1177/0049124189017003004

Bollen, K. A., & Bauldry, S. (2011). Three Cs in measurement models: Causal indicators, composite indicators, and covariates. *Psychological Methods*, *16*(3), 265–284. https://doi.org/10.1037/a0024448

Chen, F. F., West, S., & Sousa, K. (2006). A Comparison of Bifactor and Second-Order Models of Quality of Life. *Multivariate Behavioral Research*, *41*(2), 189–225. https://doi.org/10.1207/s15327906mbr4102_5

Dueber, D. M. (2017). *Bifactor Indices Calculator* (p. 473 KB) [Excel file]. University of Kentucky Libraries. https://doi.org/10.13023/EDP.TOOL.01

Fayers, P. M., & Machin, D. (2001). *Quality of life: Assessment, analysis and interpretation* (Reprint). Wiley.

Gagliardi, J., Brettschneider, C., & König, H.-H. (2021). Health-related quality of life of refugees: A systematic review of studies using the WHOQOL-Bref instrument in general and clinical refugee populations in the community setting. *Conflict and Health*, *15*(1), 44. https://doi.org/10.1186/s13031-021-00378-1

Guaracha-Basáñez, G. A., Contreras-Yáñez, I., Hernández-Molina, G., Estrada-González, V. A., Pacheco-Santiago, L. D., Valverde-Hernández, S. S., Galindo-Donaire, J. R., Peláez-Ballestas, I., & Pascual-Ramos, V. (2022). Quality of life of patients with rheumatic diseases during the COVID-19 pandemic: The biopsychosocial path. *PLOS ONE*, *17*(1), e0262756. https://doi.org/10.1371/journal.pone.0262756

Hammer, J. H., Heath, P. J., & Vogel, D. L. (2018). Fate of the total score: Dimensionality of the Conformity to Masculine Norms Inventory-46 (CMNI-46). *Psychology of Men & Masculinity*, *19*(4), 645–651. https://doi.org/10.1037/men0000147

Hancock, G. R., & Mueller, R. O. (2001). *Rethinking construct reliability within latent variable systems*. *195*(216).

Hargreaves, S. M., Nakano, E. Y., Han, H., Raposo, A., Ariza-Montes, A., Vega-Muñoz, A., & Zandonadi, R. P. (2021). Quality of Life of Brazilian Vegetarians Measured by the WHOQOL-BREF: Influence of Type of Diet, Motivation and Sociodemographic Data. *Nutrients*, *13*(8), 2648. https://doi.org/10.3390/nu13082648

Hewitt-Ramírez, N., Juárez, F., Parada-Baños, A. J., Nuñez-Estupiñán, X., & Quintero-Barrera, L. (2020). Efficacy of a primary care mental health program for victims of the armed conflict in Colombia. *Peace and Conflict: Journal of Peace Psychology*, *26*(1), 62–77. https://doi.org/10.1037/pac0000436

Hu, L., & Bentler, P. M. (1998). Fit indices in covariance structure modeling: Sensitivity to underparameterized model misspecification. *Psychological Methods*, *3*(4), 424–453. https://doi.org/10.1037/1082-989X.3.4.424

Hu, L., & Bentler, P. M. (1999). Cutoff criteria for fit indexes in covariance structure analysis: Conventional criteria versus new alternatives. *Structural Equation Modeling: A Multidisciplinary Journal*, *6*(1), 1–55. https://doi.org/10.1080/10705519909540118

Jackson, D. L., Gillaspy, J. A., & Purc-Stephenson, R. (2009). Reporting practices in confirmatory factor analysis: An overview and some recommendations. *Psychological Methods*, *14*(1), 6–23. https://doi.org/10.1037/a0014694

Kline, T. (2005). *Psychological testing: A practical approach to design and evaluation*. Sage Publications.

Lee, S.-Y., Song, X.-Y., Skevington, S., & Hao, Y.-T. (2005). Application of Structural Equation Models to Quality of Life. *Structural Equation Modeling: A Multidisciplinary Journal*, *12*(3), 435–453. https://doi.org/10.1207/s15328007sem1203_5

López Huerta, J. A., González Romo, R. A., & Tejada Tayabas, J. M. (2017). Propiedades psicométricas de la versión en español de la escala de calidad de vida WHO QOL BREF en una muestra mexicana. *Revista Iberoamericana de Diagnóstico y Evaluación - e Avaliação Psicológica*, *44*(2), 105–115. https://doi.org/10.21865/RIDEP44.2.09

Lucas-Carrasco, R. (2012). The WHO quality of life (WHOQOL) questionnaire: Spanish development and validation studies. *Quality of Life Research*, *21*(1), 161–165. https://doi.org/10.1007/s11136-011-9926-3

MacCallum, R. C. (1995). Model specification: Procedures, strategies, and related issues. In *Structural equation modeling: Concepts, issues, and applications.* (pp. 16–36). Sage Publications, Inc.

Martini, S., Setia Ningrum, D. A., Abdul-Mumin, K. H., & Yi-Li, C. (2022). Assessing quality of life and associated factors in post-stroke patients using the world health organization abbreviated generic quality of life questionnaire (WHOQOL-BREF). *Clinical Epidemiology and Global Health*, *13*, 100941. https://doi.org/10.1016/j.cegh.2021.100941

Masaeli, N., & Billieux, J. (2022). Is Problematic Internet and Smartphone Use Related to Poorer Quality of Life? A Systematic Review of Available Evidence and Assessment Strategies. *Current Addiction Reports*, *9*(3), 235–250. https://doi.org/10.1007/s40429-022-00415-w

Ohaeri, J. U., Awadalla, A. W., El-Abassi, A.-H. M., & Jacob, A. (2007). Confirmatory factor analytical study of the WHOQOL-Bref: Experience with Sudanese general population and psychiatric samples. *BMC Medical Research Methodology*, *7*(1), 37. https://doi.org/10.1186/1471-2288-7-37

Oliveira, S. E. H., Carvalho, H., & Esteves, F. (2016). Toward an understanding of the quality of life construct: Validity and reliability of the WHOQOL-Bref in a psychiatric sample. *Psychiatry Research*, *244*, 37–44. https://doi.org/10.1016/j.psychres.2016.07.007

Perera, H. N., Izadikhah, Z., O’Connor, P., & McIlveen, P. (2018). Resolving Dimensionality Problems With WHOQOL-BREF Item Responses. *Assessment*, *25*(8), 1014–1025. https://doi.org/10.1177/1073191116678925

Reise, S. P., Bonifay, W. E., & Haviland, M. G. (2013). Scoring and Modeling Psychological Measures in the Presence of Multidimensionality. *Journal of Personality Assessment*, *95*(2), 129–140. https://doi.org/10.1080/00223891.2012.725437

Rodriguez, A., Reise, S. P., & Haviland, M. G. (2016). Evaluating bifactor models: Calculating and interpreting statistical indices. *Psychological Methods*, *21*(2), 137–150. https://doi.org/10.1037/met0000045

Schermelleh-Engel, K., Moosbrugger, H., & Müller, H. (2003). Evaluating the Fit of Structural Equation Models: Tests of Significance and Descriptive Goodness-of-Fit Measures. *Methods of Psychological Research Online*, *8*(2), 52.

Simancas-Fernández, M. R., Celedón-Rivero, J. C., Salas-Picón, W. M., Salgado-Ruiz, D. E., Echeverry-Londoño, M. C., Vásquez-Campos, J. H., Guarch-Rubio, M., & Manzanero, A. L. (2021). Trauma, Reparation, and Quality of Life in Victims of Armed Conflict after Peace Agreements. *Anuario de Psicología Jurídica*, *32*(1), 79–86. https://doi.org/10.5093/apj2021a21

Singh, A., Palaniyandi, S., Palaniyandi, A., & Gupta, V. (2022). Health related quality of life among rural elderly using WHOQOL-BREF in the most backward district of India. *Journal of Family Medicine and Primary Care*, *11*(3), 1162. https://doi.org/10.4103/jfmpc.jfmpc_1073_21

Steiger, J. H. (2007). Understanding the limitations of global fit assessment in structural equation modeling. *Personality and Individual Differences*, *42*(5), 893–898. https://doi.org/10.1016/j.paid.2006.09.017

Stucky, B. D., & Edelen, M. O. (2014). Using hierarchical IRT models to create unidimensional measures from multidimensional data. In *Handbook of item response theory modeling* (pp. 201–224). Routledge, Taylor & Francis Group.

Stucky, B. D., Thissen, D., & Orlando Edelen, M. (2013). Using Logistic Approximations of Marginal Trace Lines to Develop Short Assessments. *Applied Psychological Measurement*, *37*(1), 41–57. https://doi.org/10.1177/0146621612462759

The Whoqol Group. (1998). The World Health Organization quality of life assessment (WHOQOL): Development and general psychometric properties. *Social Science & Medicine*, *46*(12), 1569–1585. https://doi.org/10.1016/S0277-9536(98)00009-4

Theuns, P., Hofmans, J., Mazaheri, M., Van Acker, F., & Bernheim, J. L. (2010). Cross-national comparability of the WHOQOL-BREF: A measurement invariance approach. *Quality of Life Research*, *19*(2), 219–224. https://doi.org/10.1007/s11136-009-9577-9

Vinaccia, S., Fernández, H., Pedro, E. M. S., & Padilla, G. V. (2006). *Aplicación de la versión española del cuestionario Quality of Life Measure for Rheumatoid Arthritis (QOL-RA) en Colombia*. *13*(4), 7.

Weston, R., & Gore, P. A. (2006). A Brief Guide to Structural Equation Modeling. *The Counseling Psychologist*, *34*(5), 719–751. https://doi.org/10.1177/0011000006286345

Whittaker, T. A. (2012). Using the Modification Index and Standardized Expected Parameter Change for Model Modification. *The Journal of Experimental Education*, *80*(1), 26–44. https://doi.org/10.1080/00220973.2010.531299

WHO. (1996). *WHOQoL-BREF Introduction, administration, scoring and generic version of the assesment*. World Health Organization.

Widaman, K. F., & Thompson, J. S. (2003). On specifying the null model for incremental fit indices in structural equation modeling. *Psychological Methods*, *8*(1), 16–37. https://doi.org/10.1037/1082-989X.8.1.16

Yao, G., Chung, C.-W., Yu, C.-F., & Wang, J.-D. (2002). Development and verification of validity and reliability of the WHOQOL-BREF Taiwan version. *Journal of the Formosan Medical Association*, *101*(5), 10.

Yuan, K.-H. (2005). Fit Indices Versus Test Statistics. *Multivariate Behavioral Research*, *40*(1), 115–148. https://doi.org/10.1207/s15327906mbr4001_5
