# Supplementary material for: Adequacy of the WHOQoL-BREF to Assess the Quality of Life of Victims of Armed Conflicts
Source: Assessment. 2023 Oct 30;31(5):1135–47. doi: 10.1177/10731911231201145 (PMC11134973; doi:10.1177/10731911231201145)
Supplement: sj-docx-2-asm-10.1177_10731911231201145 – Supplemental material for Adequacy of the WHOQoL-BREF to Assess the Quality of Life of Victims of Armed Conflicts [file sj-docx-2-asm-10.1177_10731911231201145.docx]

**Supplementary material 3**

**Table**

Comparison summary of all models. The goodness of fit indices reported are χ ^2^ (chi-squared), TLI (Tucker-Lewis Index), RMSEA (Root Mean Square Error of Approximation), CFI (Comparative Fit Index), SRMR (Standardized Root Mean Square Residual)

| Model | Description | *χ* ^2^ (df) | TLI | RMSEA (90%) | CFI | SRMR |
| --- | --- | --- | --- | --- | --- | --- |
| Original  *N = 1136* | N/A | 1260.501 (246)  *p* < 0.001 | 0.808 | 0.060  CI: [0.057-0.064] | 0.829 | 0.060 |
| Model a  *N = 1136* | *Q8* to cross load on Psychological | 1066.040 (245)  *p* < 0.001 | 0.844 | 0.054  CI: [00.51-0.058] | 0.861 | 0.051 |
| Model b  *N = 1136* | *Q9* to cross load on Psychological | 1009.346 (244)  *p* < 0.001 | 0.854 | 0.053  CI: [0.049-0.056] | 0.871 | 0.048 |
| 4F  *N = 1136* | WHO (1996)  *(With modifications)* | 628.755 (243)  *p* < 0.001 | 0.845 | 0.053  CI: [0.048-0.058] | 0.864 | 0.052 |
| 5F_R_  *N = 1136* | Oliveira et al. (2016) | 340.874 (125)  *p* < 0.001 | 0.874 | 0.055  CI: [0.048-0.062] | 0.897 | 0.044 |
| 5F  *N = 1136* | Ohaeri et al. (2007) | 696.282 (242)  *p* < 0.001 | 0.817 | 0.057  CI: [0.053-0.063] | 0.839 | 0.058 |
| Unidimensional model  *N = 1136* |  | 1107.823 (252)  *p* < 0.001 | 0.926 | 0.055  CI: [0.051-0.058] | 0.932 | 0.063 |
| Hierarchical model  *N = 1136* |  | 619.914 (246)  *p* < 0.001 | 0.967 | 0.037  CI: [0.033-0.040] | 0.970 | 0.046 |
| Bifactor model  *N = 1136* |  | 420.113 (226)  *p* < 0.001 | 0.981 | 0.028  CI: [0.022-0.030] | 0.985 | 0.038 |
